# Surrogate endpoints for overall survival in digestive oncology trials: which candidates? A questionnaires survey among clinicians and methodologists

**DOI:** 10.1186/1471-2407-10-277

**Published:** 2010-06-10

**Authors:** Nicolas Methy, Laurent Bedenne, Franck Bonnetain

**Affiliations:** 1 Fédération Francophone de Cancérologie Digestive, Inserm U866, Université de Bourgogne, Faculté de Médecine, 7 bd Jeanne d'Arc, BP 87900, 21079 Dijon Cedex, France; 2 Biostatistics and epidemiological unit, EA 4184, 1 rue Professeur Marion, BP 77980 21079 Dijon Cedex, France

## Abstract

**Background:**

Overall survival (OS) is the gold standard for the demonstration of a clinical benefit in cancer trials. Replacement of OS by a surrogate endpoint allows to reduce trial duration. To date, few surrogate endpoints have been validated in digestive oncology. The aim of this study was to draw up an ordered list of potential surrogate endpoints for OS in digestive cancer trials, by way of a survey among clinicians and methodologists. Secondary objective was to obtain their opinion on surrogacy and quality of life (QoL).

**Methods:**

In 2007 and 2008, self administered sequential questionnaires were sent to a panel of French clinicians and methodologists involved in the conduct of cancer clinical trials. In the first questionnaire, panellists were asked to choose the most important characteristics defining a surrogate among six proposals, to give advantages and drawbacks of the surrogates, and to answer questions about their validation and use. Then they had to suggest potential surrogate endpoints for OS in each of the following tumour sites: oesophagus, stomach, liver, pancreas, biliary tract, lymphoma, colon, rectum, and anus. They finally gave their opinion on QoL as surrogate endpoint. In the second questionnaire, they had to classify the previously proposed candidate surrogates from the most (position #1) to the least relevant in their opinion.

Frequency at which the endpoints were chosen as first, second or third most relevant surrogates was calculated and served as final ranking.

**Results:**

Response rate was 30% (24/80) in the first round and 20% (16/80) in the second one. Participants highlighted key points concerning surrogacy. In particular, they reminded that a surrogate endpoint is expected to predict clinical benefit in a well-defined therapeutic situation. Half of them thought it was not relevant to study QoL as surrogate for OS.

DFS, in the neoadjuvant settings or early stages, and PFS, in the non operable or metastatic settings, were ranked first, with a frequency of more than 69% in 20 out of 22 settings. PFS was proposed in association with QoL in metastatic primary liver and stomach cancers (both 81%). This composite endpoint was ranked second in metastatic oesophageal (69%), colorectal (56%) and anal (56%) cancers, whereas QoL alone was also suggested in most metastatic situations.

Other endpoints frequently suggested were R0 resection in the neoadjuvant settings (oesophagus (69%), stomach (56%), pancreas (75%) and biliary tract (63%)) and response. An unexpected endpoint was metastatic PFS in non operable oesophageal (31%) and pancreatic (44%) cancers. Quality and results of surgical procedures like sphincter preservation were also cited as eligible surrogate endpoints in rectal (19%) and anal (50% in case of localized disease) cancers. Except for alpha-FP kinetic in hepatocellular carcinoma (13%) and CA19-9 decline (6%) in pancreas, few endpoints based on biological or tumour markers were proposed.

**Conclusion:**

The overall results should help prioritise the endpoints to be statistically evaluated as surrogate for OS, so that trialists and clinicians can rely on endpoints that ensure relevant clinical benefit to the patient.

## Background

In cancer clinical trials, overall survival (OS), defined as time from randomization to death from any cause, is considered as the gold standard endpoint for the demonstration of clinical benefit [1]. It is an objective, unambiguously defined endpoint with clear clinical meaning to the patient. However, the evaluation of OS may require extended follow-up and the effect of the experimental treatment on OS may be confounded by effective subsequent therapies, including cross-over to the experimental treatment for patients initially allocated to control therapy [2].

The use of an earlier alternative endpoint would allow to shorten clinical trials and to evaluate treatment effect with better specificity. Such an endpoint can be used as definitive measure of treatment clinical efficacy instead of OS if it is *surrogate *for OS. A surrogate endpoint is defined as a biomarker "expected to predict clinical benefit (or harm or lack of benefit or harm) based on epidemiologic, therapeutic, pathophysiologic, or other scientific evidence" [3]. In addition to the biologic plausibility of the relationship between the surrogate and OS, evaluation of a surrogate endpoint requires statistical analyses using data from completed phase III clinical trials to demonstrate how reliably and in which proportion an effect on the surrogate can predict the effect on OS [4,5].

In digestive oncology, few variables have undergone evaluation process [5-7]. To date, only disease-free survival (DFS) for the adjuvant treatment of colon cancer [8,9] and progression-free survival (PFS) in the metastatic setting [10,11] have been fully validated as surrogates for OS for the evaluation of first line chemotherapies. It is therefore of interest to identify putative surrogates, so that they can get priority statistical evaluation.

The main objective of this study was to draw up a list of potential surrogate endpoints for OS in digestive oncology trials, by way of a survey among clinicians and methodologists involved in the conduct of clinical trials. Secondary objective was to obtain their opinion on particular points concerning surrogacy and quality of life (QoL).

## Methods

We polled a panel of French clinicians and methodologists involved in cancer trials by means of self administered sequential questionnaires in 2007 and 2008. The panel was composed of 14 methodologists or biostatisticians, and 66 clinicians working in the field of cancer clinical trials (gastroenterologists, oncologists, surgeons, radiotherapists). Clinicians were members of the scientific committee of the *Fédération Francophone de Cancérologie Digestive *(FFCD), to whom the protocol of the survey was presented during a scientific council sitting.

The design of our survey was based on Delphi technique guidelines [12], which is a consensus method. However, in our survey, while participants were asked to classify the endpoints they proposed, they did not re-evaluate their opinion according to responses of other panellists and formal consensus was not searched for.

**Figure 1:**
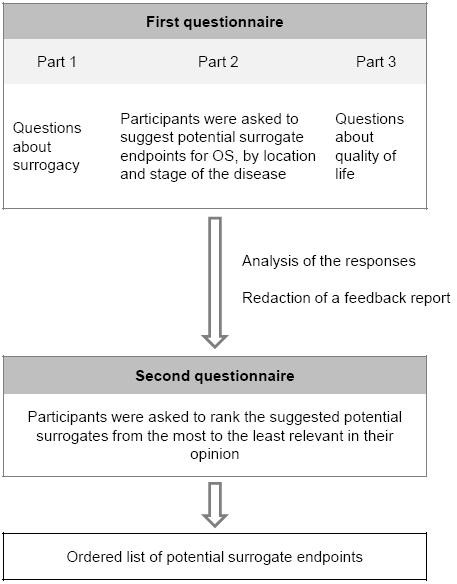
**Survey procedure**.

Two rounds were planned (Figure 1). In each round the questionnaire was sent by postal way and by e-mail. Reminder letter (e-mail) was sent about two months after the postal delivery. The second questionnaire was sent to all panellists, whether they had answered the first one or not.

First Round. The first questionnaire was composed of three parts (see additional file 1: Survey questionnaire No1):

- Part I: panellists were asked 1) to choose the most important characteristics defining a surrogate among six proposals; 2) to give advantages and drawbacks of the surrogates and 3) to answer questions about their validation and use in digestive oncology;

- Part II: they were asked to mention, if any, endpoints they thought worthwhile to be evaluated as surrogate for OS in each of the following tumour sites, according to the stage of the disease (e.g. locally advanced, metastatic): oesophagus, stomach, liver, pancreas, biliary tract, lymphoma, colon, rectum, and anus;

- Part III: they were asked to give their opinion about quality of life (QoL) as candidate surrogate.

At the end of the questionnaire, they were free to add comments.

Second Round (see additional file 2: Survey questionnaire No2):

A feedback report summarized the respondents' answers to parts I and III items and accompanied the second questionnaire. The second questionnaire tabulated all the surrogate endpoints proposed in part II of the first questionnaire. In each situation, participants were asked to classify the previously proposed surrogates, from the most promising (position #1) to the last promising in their opinion.

Descriptive statistics (histograms with frequencies) were used to summarize the responses of the first round (parts I and III). For each proposed surrogate, the frequency (%) at which it was chosen in the three first positions was calculated and served as numerical classification for the final ranking.

## Results

### First round

It was initiated in June 2007 and closed in September 2007. The response rate was 30% (24/80). Respondents were composed of 14 gastroenterologists, three oncologists, one surgeon, one radiotherapist, four methodologists and one biostatistician.

#### Part 1

**Figure 2:**
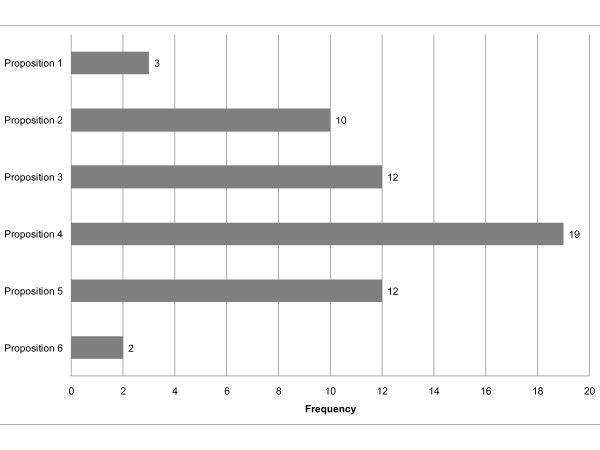
**Participant answers to the question: "The following sentences may characterize a surrogate endpoint. Which one or which ones (maximum 3) seem important for you to remember?"**. Proposition 1: "A surrogate endpoint is a variable known to be a prognostic factor" Proposition 2: "A surrogate endpoint is a variable correlated with overall survival" Proposition 3: "A surrogate endpoint is a biomarker^1 ^that is intended to substitute for a clinical endpoint. A surrogate endpoint is expected to predict clinical benefit (or harm or lack of benefit or harm) based on epidemiologic, therapeutic, pathophysiologic, or other scientific evidence" (ref. [3]) ^1 ^A biomarker is "a characteristic that is objectively measured and evaluated as an indicator of normal biological processes, pathogenic processes, or pharmacologic responses to a therapeutic intervention" Proposition 4: "A surrogate endpoint is an intermediate endpoint, reliably and reproducibly measurable (imaging, biology...), which allows to predict the effect of the treatment on the clinical endpoint" (ref. [13]) Proposition 5: "A surrogate endpoint is a variable which is accepted as primary endpoint in clinical trials by medical agencies (e.g. FDA, EMEA, AFSSAPS, etc.)^2^" ^2 ^FDA, (United State) Food and Drug Administration; EMEA, European Medicines Agency; AFSSAPS, Agence Française de Sécurité Sanitaire des Produits de Santé Proposition 6: "A surrogate endpoint is a response variable for which a test of the null hypothesis of no relationship to the treatment groups under comparison is also a valid test of the corresponding null hypothesis based on the true endpoint" (ref. [14])

The most frequently chosen sentence among the six proposed to characterize a surrogate endpoint was the one that defined a surrogate as "an intermediate variable, reliably and reproducibly measurable, which allows to predict the effect of treatment on the clinical endpoint" [13] (chosen by 19 participants out of 24). Definition of surrogacy in terms of the equivalence of hypothesis tests for the treatment effects (Prentice's definition [14]) was the less chosen (2 out of 24) (Figure 2).

Participants underlined several advantages and risks or limitations in using surrogate endpoints. On the one hand, they said that surrogates may allow to reduce trial duration, the number of patients and/or the cost of the studies, and to avoid the confounding effect of subsequent effective therapies. On the other hand, they questioned their validity and warned from the risk of erroneous conclusions. They also set limits to generalization of the results to other settings than the one used for validation (Table 1).

**Table 1:** Advantages and risks or limitations of surrogate endpoints put forward by the participants.

**Advantages**	**Risks or limitations**
To reduce trial duration • Accelerate approval and dissemination of effective therapies • More rapidly pass to another question	Use of non validated surrogates Generalization of the results to another setting or patient population
To decrease the required number of patients	Alteration of the validity after therapeutic advances
To decrease the cost of the trial	Risk of erroneous conclusion concerning survival (reliability), late toxicity not measured
Endpoints not confounded by subsequent lines of treatment • Better imputability • Power (sensibility) gain	Limitations regarding the definition and the reproducibility of the surrogate endpoint
Relevance of the surrogate endpoint in itself	Cost

**Figure 3:**
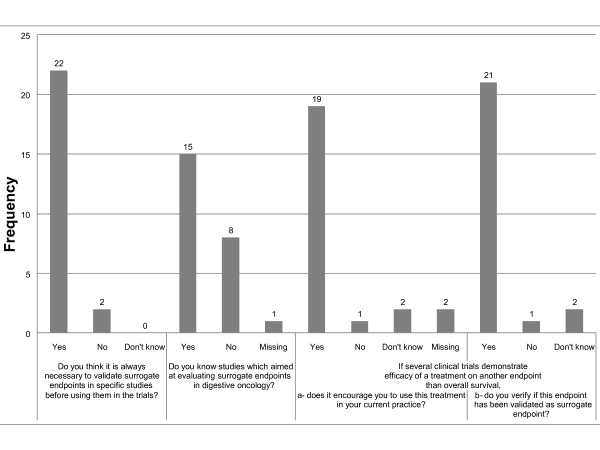
**Participant answers to questions about validation and use of surrogate endpoints**.

Twenty two out of 24 participants thought it was necessary to perform specific validation studies before using a surrogate endpoint, and fifteen stated to have knowledge of such studies. They quoted Buyse *et al*. 2000 [15] and Johnson *et al*. 2006 [16], for advanced colorectal cancer, and Sargent *et al*. 2005 [8] in colorectal cancer adjuvant setting. They also quoted the ongoing meta-analysis GASTRIC [17] which is aimed at evaluating DFS and PFS in stomach cancers. The large majority of participants wrote they were ready to rely on other endpoints than OS (19/24) after having verified their validation as surrogates for OS (21/24) (Figure 3).

#### Part 2

Endpoints proposed for surrogacy evaluation were resituated in the second questionnaire (see Second round paragraph and additional file 3: Propositions and rankings of potential surrogate endpoints for overall survival). To the question "Are there situations in which you think looking for surrogate endpoints is useless?" some respondents answered bad prognostic cancers such as pancreas and oesophagus metastatic cancers, whereas others thought it was always useful.

#### Part 3

**Figure 4:**
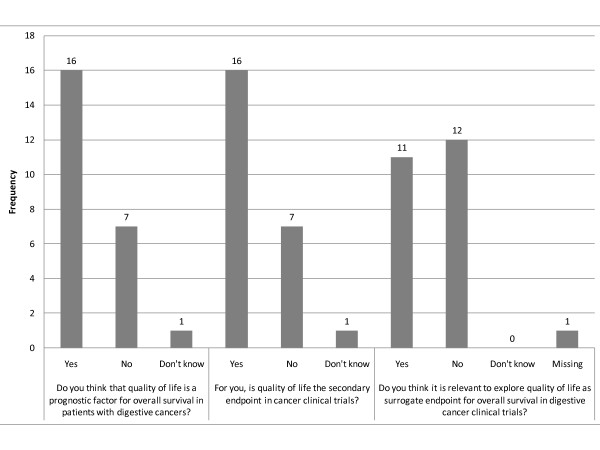
**Participant answers to questions about quality of life**.

Two thirds of the participants thought that QoL was both a prognostic factor and the secondary endpoint in digestive oncology clinical trials, whereas only half of them thought it would be relevant to evaluate its surrogacy (Figure 4).

At the end of the questionnaire, free remarks concerned QoL. It was said to be too much difficult to be quantified and too much treatment period dependent. QoL was said to be relevant for bad prognostic cancers, for which a benefit of QoL could be preferred to a survival gain. Other participants stated that QoL was a relevant outcome on its own merit.

### Second round

It was initiated in July 2008 and closed in October 2008. The response rate was 20% (16/80). Respondents were composed of nine gastroenterologists, two oncologists, two surgeons, one radiotherapist, one methodologist and one statistician. Ten of these respondents had answered the first questionnaire.

For each cancer site and stage, final ranking of the endpoints was performed according to the frequency at which they were chosen as first, second or third most relevant potential surrogates by each participant (for complete detailed results, see additional file 3: Propositions and rankings of potential surrogate endpoints for overall survival). In oesophagus cancer, preferred endpoints in the neoadjuvant setting were DFS, R0 resection and response (81%, 69%, 44%, respectively). In case of non operable, non metastatic disease, PFS (69%), response (63%), DFS (50%) and metastatic PFS (31%) were ranked number 1, 2 and 3, respectively. In the metastatic setting, PFS, QoL in association with PFS, and QoL alone were the best rated endpoints (69%, 63%, 44%, respectively). Other endpoints proposed for oesophagus cancer included metabolic response, ratio involved nodes to examined nodes and dysphasia-free survival.

In stomach cancer, preferred potential surrogate endpoints in the neoadjuvant setting were DFS (93%), R0 resection (56%) and response (44%). In the metastatic setting, QoL associated with PFS was ranked first (81%), followed by PFS alone (75%), response and QoL alone (both 44%).

For small hepatocellular carcinoma (HCC), DFS, PFS, local control and response were the preferred potential surrogate endpoints (69%, 44%, 31%, 31%, respectively). In case of advanced HCC, preferred endpoints were PFS (88%), response (50%) and QoL (44%). For metastatic disease, QoL in association with PFS was ranked first (81%), followed by PFS alone (56%) and QoL alone (31%). Other endpoints included hospitalisation-free survival, hepatocellular function, clinical benefit and alpha-FP kinetic.

In pancreas cancer, best rated endpoints in the neoadjuvant setting were DFS (88%) and R0-R1 resection. For non operable tumours, PFS (88%), QoL (50%) and metastatic PFS (44%) were the best rated endpoints. For metastatic disease, PFS was ranked first (81%), followed by symptom-free survival (50%). Other endpoints for pancreas cancer included response, hospitalisation-free survival, CA19-9 decline and pain.

For biliary tract cancer, best rated endpoints in the neoadjuvant setting were DFS (81%) and R0 resection (63%). For non operable, non metastatic disease, preferred endpoints were PFS (81%), QoL (63%) and response (56%). In the metastatic setting, preferred endpoints were PFS, response and QoL (75%, 56%, 50%, respectively). Icterus-free survival was among the other suggested endpoints.

For localized digestive lymphoma, preferred endpoints were DFS and response (69%, 31%, respectively). Other endpoints included percentage of high-grade transformation per year and gastrectomy avoidance. In the metastatic setting, DFS was ranked first (63%). Other endpoints included response (38%) and time-to-remission (38%).

For the adjuvant treatment of colon cancer, participants preferred DFS (93%), specific survival (56%) and QoL (31%). For the neoadjuvant treatment of rectal cancer, DFS was ranked first (100%), followed by response, complete resection, sphincter preservation and QoL (69%, 56%, 19%, 6%, respectively). In metastatic colorectal cancer, best rated endpoints were PFS (75%), QoL in association with PFS, and R0 metastatic resection rate (both 56%). Other endpoints included response, QoL, cumulative time without cytotoxic treatment and maintenance regimen-free survival.

For non metastatic anal cancer, preferred endpoints were DFS, response, sphincter preservation rate and abdominoperitoneal amputation-free survival (69%, 50%, 50%, 31%, respectively for localized disease, and 50%, 31%, 25%, 25%, respectively for locally advanced disease). In the metastatic setting, PFS was ranked first (69%), followed by QoL in association with PFS (56%). Other endpoints included symptom-free survival, response and QoL.

## Discussion

Participants of our survey highlighted key points of the definition, properties and use of surrogate endpoints for OS in digestive cancer clinical trials. Because other criteria than OS take part in their therapeutic decisions and practice, they agreed on the need for validation, to ensure that surrogate endpoints allow reliable prediction of survival benefit. A large amount of endpoints was proposed for surrogacy evaluation. Event-free survival endpoints, such as DFS and PFS, were preferred to early treatment effect evaluation, such as R0 resection and response. There was discrepancy about the relevance of studying QoL as surrogate for OS. However, QoL was often proposed in metastatic stages, in association or not with PFS.

Our panellists were active participants in the field of oncology trials (investigators, methodologists), therefore the results of this survey may not fully reflect knowledge and opinions of other professionals not directly involved in the conduct of a trial. Indeed, we limited our panel to FFCD scientific council members and methodologists in anticancer centres for practical reasons, but also under the assumption that they would be more committed and disposed to participate. However, a low proportion of them fulfilled the questionnaires. One can suppose that some non-respondents felt not sufficiently comfortable with this issue. Besides, the parts of the questionnaires that were asking participants to suggest and classify potential surrogate endpoints may have best suited clinicians than methodologists. Moreover, since a lot of digestive tumour sites and settings were dealt with, the questionnaires may have appeared heavy-going. A higher participation rate could have generated more propositions of potential surrogate endpoints and might have modified the final sorting. The survey was also performed with an educational goal, to lead people to question themselves on the relevance of the endpoints used in oncology trials. Thus we chose a self-administered questionnaires approach to allow participants to freely express their opinion, without being influenced by others.

Respondent answers covered major issues surrounding surrogacy. The main point concerns the difference with a prognostic factor. Though most surrogate endpoints are also prognostic, a prognostic factor is not necessarily a surrogate [18,19]: "a correlate does not a surrogate make" [20]. Indeed, even if two *endpoints *are correlated, *treatment effects *on them are not necessarily correlated too and effect on an intermediate variable does not necessarily predict the effect on the final endpoint [20]. Half of our participants related surrogacy with outcomes accepted as primary efficacy endpoints for the evaluation of oncology products by medical agencies, such as the US Food and Drug Administration and the European Agency for the Evaluation of Medicinal Products. They actually both may rely on surrogate endpoints for marketing approvals [21,22]. A *well-established *(validated) surrogate would be able to support regular approval, whereas a surrogate "*reasonably likely to predict clinical benefit*" might only support accelerated/conditional approvals [23,24]. Because of the difficulty in establishing reliable surrogacy, debates still remain around the use of surrogate endpoints as definitive efficacy criteria [25,26]. Our participants underlined some risks and limitations. They reminded that surrogacy depends upon the disease setting and the class of therapeutic agents and that validation cannot be extrapolated to another situation without further investigation. They added that there may still remain a risk of erroneous conclusion on OS and that short follow-up does not allow to observe unintended late adverse events. The established surrogate association may also alter over time with diagnosis and therapeutic advances, as discussed for 3-year DFS in adjuvant colon cancer trials [8,9,27,28].

DFS and PFS have been ones of the preferred candidate surrogate endpoints, whatever cancer site. This could be explained by the fact that both have been validated and used in colorectal cancer trials. DFS, defined as the time from randomization to the first event of either recurrent disease or death (second primary tumours not counted as events) has been validated as surrogate for OS in adjuvant colon cancer trials [8,9]. PFS, defined as the time from random assignment to progressive disease or death from any cause, has been validated in advanced colorectal cancer [10,11]. On the contrary, time-to-progression, which does not include death as an event contrary to PFS, was shown to be less reliable [10,16]. As for response rate, while allowing an early evaluation of treatment effect, it does not allow accurate prediction of OS in colorectal cancers [6,10,15,16].

DFS and PFS were sometimes both proposed in the questionnaire for the same disease stage. In accordance with Delphi guidelines, which advise to keep all participant propositions from first to second round [12], we let both endpoints in the second questionnaire. However, it is finally unclear regarding the events taken into account. This raises the preliminary question of the definition of the survival endpoints, for which a uniform terminology has been stressed as a main concern to compare trial results [29]. In digestive oncology, standardization of the definitions of such endpoints has been carried out for hepatocellular carcinoma trials [30] and colon cancer trials [31,32]. In order to reach a consensus in oncology trials, a European Delphi survey has been initiated by French methodologists and statisticians from oncology centres and from the FFCD, in collaboration with the European Organisation for Research and Treatment of Cancer. Secondary objectives of that study are to assess the influence of the definitions over trial results and to investigate their surrogacy.

In some situations, OS is no longer a reliable endpoint, e.g. in advanced colorectal cancer treated in the first line, mainly because of the increasing availability of effective agents in the subsequent lines [33]. In such cases, rather than or before dealing with surrogacy issue, one has to define the most appropriate clinical efficacy endpoint. Clinical value to the patient of endpoints other than OS is thus sometimes questioned. This has been discussed for PFS in advanced colorectal cancer [25,33] and for DFS in adjuvant trials, where disease recurrence has high subsequent costs, QoL impact and debilitating consequences [8]. Similarly, in rectal cancer, preoperative chemoradiation has become the standard neoadjuvant treatment because of a significant benefit with respect to local control compared with preoperative radiotherapy alone, though no impact on survival was demonstrated [34,35].

As reminded by Fleming *et al*. [25], "oncology patients are interested in achieving clinically meaningful beneficial effects on disease-related symptoms, on ability to carry out normal activities, and on OS". As a direct measure of how a patient feels, functions, or survives [3], QoL is considered as a clinical endpoint, thus allowing QoL improvement alone to serve as a basis for marketing approval [36]. By definition, QoL would be additionally surrogate for OS if treatment effect on QoL predicted a survival benefit. While our participants were divided about studying QoL as surrogate for OS in the first round, QoL was generally well-rated in the second round for advanced stages. They put forward complementarity between PFS and QoL by proposing these criteria as a composite surrogate endpoint for OS. Two thirds of our participants declared they considered QoL as the secondary endpoint in cancer clinical trials. There is an increasing interest in endpoints such as time to symptomatic deterioration or time to QoL deterioration. However, the variability in the definition and analysis of QoL endpoints, including rules about censoring and missing data, cut-off for QoL decrease, and QoL dimensions, currently limit surrogacy validation. Nevertheless, simple but well-validated tools for the measurement of global QoL [37] could be incorporated into future trials with relative ease for surrogacy evaluation purpose.

## Conclusions

In conclusion, the overall results of our study should help prioritise the endpoints to be statistically evaluated as surrogate for OS and highlight original potential alternative endpoint. As preferred potential surrogates in each digestive tumour site, event-free survival endpoints should be first evaluated, with particular interest for QoL or composite endpoints including QoL in the metastatic setting.

## List of abbreviations

DFS: disease-free survival; FFCD: Fédération Francophone de Cancérologie Digestive; OS: overall survival, PFS: progression-free survival; QoL: quality of life.

## Competing interests

The authors declare that they have no competing interests.

## Authors' contributions

FB conceived of the study. NM, LB, FB participated in the design of the study. NM performed the statistical analysis and wrote the manuscript. FB, LB helped to draft the manuscript. All authors read and approved the final manuscript.

## Pre-publication history

The pre-publication history for this paper can be accessed here:

http://www.biomedcentral.com/1471-2407/10/277/prepub

## Supplementary Material

Additional file 1 **Survey questionnaire No1**. Click here for file

Additional file 2 **Survey questionnaire No2**. Click here for file

Additional file 3 **Propositions and rankings of potential surrogate endpoints for overall survival**. In each situation, participants classified the surrogates from the most (#1) to the least relevant for surrogacy evaluation in their opinion, or checked "Do not study". Endpoints are classified according to the number of times they were chosen in first, second or third position. Click here for file
