# Supplementary material for: Surrogate endpoints for overall survival in digestive oncology trials: which candidates? A questionnaires survey among clinicians and methodologists
Source: BMC Cancer. 2010 Jun 10;10:277. doi: 10.1186/1471-2407-10-277 (PMC2904280; doi:10.1186/1471-2407-10-277)
Supplement: Additional file 1 — Survey questionnaire No1. [file 1471-2407-10-277-S1.PDF]

**Enquête Delphi**	**Critères de substitution *(surrogate endpoints)* dans les essais cliniques de cancérologie digestive**

PREMIER TOUR

Questionnaire à retourner à

sous version papier :
Nicolas Méthy
FFCD – Faculté de Médecine
7 bd Jeanne d'Arc – BP 87900
21079 DIJON Cedex

sous version électronique :
nicolas.methy@u-bourgogne.fr

NOM **PRENOM**

NB : les réponses seront traitées anonymement

SPECIALITE

☐ Gastroentérologue ☐ Oncologue ☐ Chirurgien ☐ Radiothérapeute
☐ Biostatisticien ☐ Méthodologiste ☐ Autre, précisez

LIEU D'EXERCICE

☐ CHU ☐ CH ☐ Centre de lutte contre le cancer
☐ Secteur libéral ☐ Autre, précisez

---

1. La notion de critère de substitution *(surrogate endpoint)*

1.1. Les propositions suivantes caractérisent un critère de substitution. Laquelle ou lesquelles (maximum 3) vous semble(nt) importante(s) à retenir ?
NB : Il n'y a pas de "mauvaise" réponse.

a. ☐ Un critère de substitution est une variable connue pour être un facteur pronostique

b. ☐ Un critère de substitution est une variable corrélée à la survie globale

c. ☐ Un critère de substitution est un biomarqueur[1] qui peut se substituer au critère de jugement clinique. Selon des arguments épidémiologiques, thérapeutiques, physiopathologiques ou d'autres arguments scientifiques, un critère de substitution doit permettre de prédire le bénéfice (ou risque) clinique (ou l'absence de bénéfice ou de risque)

[1] Un biomarqueur est une caractéristique mesurée objectivement qui est un indicateur d'un processus biologique normal ou pathologique, ou de la réponse à une intervention thérapeutique

d. ☐ Un critère de substitution est un critère intermédiaire mesurable de façon fiable et reproductible (imagerie, biologie...) qui permet de prédire l'effet d'un traitement sur le critère clinique

e. ☐ Un critère de substitution est une variable acceptée par les autorités de santé (FDA, EMEA, AFSSAPS, etc.)[2] comme critère de jugement principal dans les essais thérapeutiques

[2] FDA : (United State) Food and Drug Administration, EMEA : European Medicines Agency, AFSSAPS : Agence Française de Sécurité Sanitaire des Produits de Santé

f. ☐ Un critère de substitution est une variable S pour laquelle tester l'hypothèse nulle "S n'est pas associée au traitement" revient à tester l'hypothèse nulle "la survie globale n'est pas associée au traitement"

Auriez-vous une définition à proposer ?

1.2. Quels avantages voyez-vous à l'utilisation de critères de substitution de la survie globale dans les essais cliniques de cancérologie digestive ?

1.3. Quels risques ou limites voyez-vous à l'utilisation de critères de substitution de la survie globale dans les essais cliniques de cancérologie digestive ?

1.4. Pensez-vous qu'il soit toujours nécessaire de valider les critères de substitution par des études *spécifiques* avant qu'ils soient utilisés dans les essais ?

☐ Oui
☐ Non
☐ NSP

1.5. Avez-vous connaissance d'études ayant pour objectif d'évaluer des critères de substitution en cancérologie digestive ?

☐ Oui
☐ Non

Si oui, précisez si possible pour quelles localisations et/ou les références.

1.6. Si plusieurs essais cliniques démontrent l'efficacité d'un traitement sur un *critère de jugement autre que la survie globale*,

a. Cela vous encourage-t-il à utiliser le traitement dans la pratique quotidienne ?

☐ Oui
☐ Non
☐ NSP

b. Vérifiez-vous si ce critère de jugement a été validé en tant que critère de substitution ?

☐ Oui
☐ Non
☐ NSP

2. Critères de substitution dans les essais cliniques de cancérologie digestive

2.1. Pour chacune des localisations cancéreuses et des stades listés dans le tableau suivant, proposez des critères qu'il vous semblerait intéressant d'évaluer comme critères de substitution de la survie globale.

**Localisation cancéreuse**<br>stade	Propositions de critères de substitution candidats<br>(en précisant éventuellement après quel traitement)
**Œsophage**	
localisé	
localement avancé	
métastatique	
**Estomac**	
non métastatique	
métastatique	
**Foie**	
petit CHC (critère de Milan)	
gros CHC	
métastatique	
**Pancréas**	
localisé	
localement avancé	
métastatique	
**Voies biliaires**	
opérable	
non opérable,<br>non métastatique	
métastatique	

**Localisation cancéreuse** stade	Propositions de critères de substitution candidats (en précisant éventuellement après quel traitement)
**Lymphomes digestifs**	
localisé	
métastatique	
**Côlon**	
stade I	
stade II	
stade III	
**Rectum**	
stade I	
stade II	
stade III	
**Côlon-rectum**	
métastatique	
**Anus**	
localisé	
localement avancé	
métastatique	

2.2. Y a-t-il des localisations et des stades pour lesquels il ne vous paraît pas utile de rechercher des critères de substitution ?

3. Qualité de vie

3.1. Pensez-vous que la qualité de vie soit un facteur pronostique de la survie globale de patients atteints d'un cancer digestif ?

- ☐ Oui
- ☐ Non
- ☐ NSP

3.2. Pour vous, la qualité de vie est-elle le second critère de jugement après la survie globale dans les essais cliniques en cancérologie ?

- ☐ Oui
- ☐ Non
- ☐ NSP

3.3. Pensez-vous qu'il soit pertinent d'explorer la qualité de vie comme critère de substitution de la survie globale dans les essais cliniques en cancérologie digestive ?

- ☐ Oui
- ☐ Non
- ☐ NSP

4. Remarques libres sur la thématique
