# Supplementary material for: Surrogate endpoints for overall survival in digestive oncology trials: which candidates? A questionnaires survey among clinicians and methodologists
Source: BMC Cancer. 2010 Jun 10;10:277. doi: 10.1186/1471-2407-10-277 (PMC2904280; doi:10.1186/1471-2407-10-277)
Supplement: Additional file 2 — Survey questionnaire No2. [file 1471-2407-10-277-S2.PDF]

Critères de substitution *(surrogate endpoints)* dans les essais cliniques de cancérologie digestive

DEUXIEME TOUR DE L'ENQUETE

Questionnaire à retourner à

sous version papier :
Nicolas Methy
FFCD – Faculté de Médecine
7 bd Jeanne d'Arc – BP 87900
21079 DIJON Cedex

sous version électronique :
nicolas.methy@u-bourgogne.fr

NOM **PRENOM**

NB : les réponses seront traitées anonymement

Pour chacune des situations suivantes, et selon votre opinion, classez en les numérotant (1, 2, 3 ...) les critères de substitution proposés, du plus pertinent (rang 1) au moins prometteur. Cochez la case *Ne pas étudier* pour ceux que vous ne retenez pas.

Pour les critères tels que survie sans récidive ou survie sans progression, précisez si possible les événements que vous considérez, comme le décès et les seconds cancers par exemple.

Situation 1. Œsophage - opérable et traitement néo-adjuvant

**Critère de substitution de la survie globale pour évaluer l'effet d'un traitement**	**Classement**<br>Rang de pertinence (1, 2, 3 ...)	*Ne pas étudier (cocher la case)*	**Précisions éventuelles**
**Réponse**	☐	☐	☐ Réponse préopératoire<br>☐ pCR (% de réponses complètes)<br>☐ Degré de réponse histologique (Mandard score)
**Chirurgie R0**	☐	☐	
**Survie sans récidive**	☐	☐	
**Survie sans progression**	☐	☐	
**Réponse métabolique précoce**	☐	☐	
**Ratio nb gg envahis / nb gg prélevés**	☐	☐	

Situation 2. Œsophage - non opérable, non métastatique

**Critère de substitution de la survie globale pour évaluer l'effet d'un traitement**	**Classement**<br>Rang de pertinence (1, 2, 3 ...)	ou	*Ne pas étudier (cocher la case)*	**Précisions éventuelles**
**Réponse (évaluation clinico-radiologique)**	☐		☐	
**Survie sans récidive**	☐		☐	
**Survie sans progression**	☐		☐	
**Survie sans progression métastatique**	☐		☐	
**Survie sans dysphagie**	☐		☐	
**Réponse métabolique**	☐		☐	

Situation 3. Œsophage - métastatique

**Critère de substitution de la survie globale pour évaluer l'effet d'un traitement**	**Classement**		**Précisions éventuelles**
	Rang de pertinence (1, 2, 3 ...)	*Ne pas étudier (cocher la case)*	
**Contrôle de symptômes spécifiques ("clinical benefit")**	☐	☐	
**Survie sans progression**	☐	☐	
**Réponse métabolique**	☐	☐	
**Réponse**	☐	☐	☐ Taux de réponses (RECIST)<br>☐ Réponse maximale à la première ligne de CT (RECIST)
**Qualité de vie**	☐	☐	
**Qualité de vie + survie sans progression (critère composite)**	☐	☐	

Situation 4. Estomac - non métastatique, opérable et traitement néo-adjuvant

**Critère de substitution de la survie globale pour évaluer l'effet d'un traitement**	**Classement**		**Précisions éventuelles**
	Rang de pertinence (1, 2, 3 ...)	*Ne pas étudier (cocher la case)*	
**Chirurgie R0**	☐	☐	
**Survie sans récidive**	☐	☐	
**Survie sans progression**	☐	☐	
**Réponse**	☐	☐	☐ Réponse préopératoire<br>☐ pCR (% de réponses complètes)

Situation 5. Estomac - métastatique

**Critère de substitution de la survie globale pour évaluer l'effet d'un traitement**	**Classement**		**Précisions éventuelles**
	Rang de pertinence (1, 2, 3 ...)	*Ne pas étudier (cocher la case)*	
**Qualité de vie**	☐	☐	
**Qualité de vie + survie sans progression (critère composite)**	☐	☐	
**Survie sans progression**	☐	☐	
**Contrôle de symptômes spécifiques ("clinical benefit")**	☐	☐	
**Réponse**	☐	☐	☐ Taux de réponses (RECIST)<br>☐ Réponse maximale à la première ligne de CT (RECIST)

Situation 6. Foie - petit CHC (critère de Milan)

**Critère de substitution de la survie globale pour évaluer l'effet d'un traitement**	**Classement**<br>Rang de pertinence (1, 2, 3 ...)	ou	*Ne pas étudier (cocher la case)*	**Précisions éventuelles**
**Survie sans récidive**	☐		☐	
**Survie sans progression**	☐		☐	
**Contrôle local**	☐		☐	
**Réponse**	☐		☐	
**Survie sans hospitalisation**	☐		☐	

Situation 7. Foie - gros CHC (critère de Milan)

**Critère de substitution de la survie globale pour évaluer l'effet d'un traitement**	**Classement**<br>Rang de pertinence (1, 2, 3 ...)	ou	*Ne pas étudier (cocher la case)*	**Précisions éventuelles**
**Fonction hépatocellulaire**	☐		☐	
**Contrôle de symptômes spécifiques ("clinical benefit")**	☐		☐	
**Survie sans hospitalisation**	☐		☐	
**Réponse**	☐		☐	
**Qualité de vie**	☐		☐	
**Survie sans progression**	☐		☐	

Situation 8. Foie - métastatique

**Critère de substitution de la survie globale pour évaluer l'effet d'un traitement**	**Classement**<br>Rang de pertinence (1, 2, 3 ...)	*Ne pas étudier (cocher la case)*	**Précisions éventuelles**
**Réponse**	☐	☐	☐ Taux de réponses (RECIST)<br>☐ Réponse aux anti-angiogéniques évaluée par imagerie
**Survie sans progression**	☐	☐	
**Qualité de vie**	☐	☐	
**Qualité de vie + survie sans progression**	☐	☐	
**Survie sans hospitalisation**	☐	☐	
**Contrôle de symptômes spécifiques ("clinical benefit")**	☐	☐	
**Cinétique évolution alpha FP (si ↑)**	☐	☐	

Situation 9. Pancréas - localisé, opérable, traitement néo-adjuvant

**Critère de substitution de la survie globale pour évaluer l'effet d'un traitement**	**Classement**<br>Rang de pertinence (1, 2, 3 ...)	ou	*Ne pas étudier (cocher la case)*	**Précisions éventuelles**
**Survie sans récidive**	☐		☐	
**Survie sans progression**	☐		☐	
**Survie sans hospitalisation**	☐		☐	
**Réponse**	☐		☐	
**Taux de résection R0 R1**	☐		☐	

Situation 10. Pancréas - localement avancé, non opérable

**Critère de substitution de la survie globale pour évaluer l'effet d'un traitement**	**Classement**<br>Rang de pertinence (1, 2, 3 ...)	*Ne pas étudier (cocher la case)*	**Précisions éventuelles**
**Réponse**	☐	☐	☐ Réponse complète<br>☐ Taux de réponses objectives
**Survie sans récidive**	☐	☐	
**Contrôle de symptômes spécifiques ("clinical benefit")**	☐	☐	
**Survie sans hospitalisation**	☐	☐	
**Survie sans progression**	☐	☐	
**Survie sans progression métastatique**	☐	☐	
**Qualité de vie**	☐	☐	

Situation 11. Pancréas - métastatique

**Critère de substitution de la survie globale pour évaluer l'effet d'un traitement**	**Classement**<br>Rang de pertinence (1, 2, 3 ...)	ou	*Ne pas étudier (cocher la case)*	**Précisions éventuelles**
**Survie sans progression**	☐		☐	
**Réponse**	☐		☐	
**Survie sans symptôme**	☐		☐	
**Contrôle de symptômes spécifiques ("clinical benefit")**	☐		☐	
**Chute CA19-9 >50%**	☐		☐	
**Qualité de vie**	☐		☐	
**Survie sans hospitalisation**	☐		☐	
**Douleurs**	☐		☐	

Situation 12. Voies biliaires - opérable, traitement néo-adjuvant

**Critère de substitution de la survie globale pour évaluer l'effet d'un traitement**	**Classement**<br>Rang de pertinence (1, 2, 3 ...)	ou	*Ne pas étudier (cocher la case)*	**Précisions éventuelles**
**Survie sans récidive**	☐		☐	
**Survie sans progression**	☐		☐	
**Survie sans hospitalisation**	☐		☐	
**Taux de résection R0**	☐		☐	
**Survie sans ictère**	☐		☐	

Situation 13. Voies biliaires - non opérable, non métastatique

**Critère de substitution de la survie globale pour évaluer l'effet d'un traitement**	**Classement**<br>Rang de pertinence (1, 2, 3 ...)	*Ne pas étudier (cocher la case)*	**Précisions éventuelles**
**Survie sans progression**	☐	☐	
**Qualité de vie**	☐	☐	
**Réponse**	☐	☐	☐ Réponse complète<br>☐ Taux de réponses objectives
**Survie sans hospitalisation**	☐	☐	
**Survie sans ictère**	☐	☐	

Situation 14. Voies biliaires - métastatique

**Critère de substitution de la survie globale pour évaluer l'effet d'un traitement**	**Classement**<br>Rang de pertinence (1, 2, 3 ...)	ou	*Ne pas étudier (cocher la case)*	**Précisions éventuelles**
**Taux de réponses objectives**	☐		☐	
**Qualité de vie**	☐		☐	
**Survie sans progression**	☐		☐	
**Survie sans hospitalisation**	☐		☐	
**Contrôle de symptômes spécifiques ("clinical benefit")**	☐		☐	

Situation 15. Lymphomes digestifs - localisé

**Critère de substitution de la survie globale pour évaluer l'effet d'un traitement**	**Classement**<br>Rang de pertinence (1, 2, 3 ...)	*Ne pas étudier (cocher la case)*	**Précisions éventuelles**
**Qualité de vie**	☐	☐	
**Survie sans récidive**	☐	☐	
**Survie sans progression**	☐	☐	
**% de transformation en haut grade / an**	☐	☐	
**Réponse**	☐	☐	☐ Réponse complète biologique<br>☐ Réponse complète histologique<br>☐ Taux de réponse
**Absence de gastrectomie**	☐	☐	

Situation 16. Lymphomes digestifs - métastatique

**Critère de substitution de la survie globale pour évaluer l'effet d'un traitement**	**Classement**<br>Rang de pertinence (1, 2, 3 ...)	*Ne pas étudier (cocher la case)*	**Précisions éventuelles**
**Survie sans progression**	☐	☐	
**Survie sans maladie**	☐	☐	
**Réponse**	☐	☐	☐ Réponse complète biologique<br>☐ Degré de réponse métabolique<br>☐ Taux de réponses objectives
**Temps jusqu'à rémission**	☐	☐	

Situation 17. Côlon - stade II-III, traitement adjuvant

**Critère de substitution de la survie globale pour évaluer l'effet d'un traitement**	**Classement**			**Précisions éventuelles**
	Rang de pertinence (1, 2, 3 ...)	ou	*Ne pas étudier (cocher la case)*	
**Survie sans récidive**	☐		☐	
**Survie sans progression**	☐		☐	
**Survie spécifique**	☐		☐	
**Qualité de vie**	☐		☐	

Situation 18. Rectum - stades II et III, traitement néo-adjuvant

**Critère de substitution de la survie globale pour évaluer l'effet d'un traitement**	**Classement**		**Précisions éventuelles**
	Rang de pertinence (1, 2, 3 ...)	*Ne pas étudier (cocher la case)*	
**Survie sans récidive**	☐	☐	☐ Récidives locales<br>☐ Récidives à distance<br>☐ Récidives locales + à distance
**Qualité de vie**	☐	☐	
**Taux de résection complète**	☐	☐	
**Réponse**	☐	☐	☐ Réponse préopératoire<br>☐ pCR<br>☐ Degré de réponse histologique
**Préservation sphinctérienne**	☐	☐	

Situation 19. Côlon-Rectum - métastatique

**Critère de substitution de la survie globale pour évaluer l'effet d'un traitement**	**Classement**		**Précisions éventuelles**
	Rang de pertinence (1, 2, 3 ...)	*Ne pas étudier (cocher la case)*	
**Survie sans progression**	☐	☐	
**Temps cumulé sans traitement cytotoxique**	☐	☐	
**Dosage des marqueurs tumoraux précoce à 1 mois**	☐	☐	
**Survie sans traitement d'entretien**	☐	☐	
**Réponse**	☐	☐	☐ Réponse tumorale<br>☐ Taux de réponse (RECIST)<br>☐ Réponse métabolique
**Qualité de vie**	☐	☐	
**Survie sans progression + qualité de vie**	☐	☐	
**Ratio survie globale / durée de chimio**	☐	☐	
**Taux de résection R0 de métastase**	☐	☐	

Situation 20. Anus - localisé

**Critère de substitution de la survie globale pour évaluer l'effet d'un traitement**	**Classement**		**Précisions éventuelles**
	Rang de pertinence (1, 2, 3 ...)	*Ne pas étudier (cocher la case)*	
**Taux de conservation sphinctérienne**	☐	☐	
**Survie sans récidive**	☐	☐	
**Survie sans progression**	☐	☐	
**Réponse**	☐	☐	
**Survie sans amputation abdominopéritonéale**	☐	☐	

Situation 21. Anus - localement avancé

**Critère de substitution de la survie globale pour évaluer l'effet d'un traitement**	**Classement** Rang de pertinence (1, 2, 3 ...)	*Ne pas étudier (cocher la case)*	**Précisions éventuelles**
**Réponse**	☐	☐	
**Survie sans amputation abdominopéritonéale**	☐	☐	
**Contrôle de symptômes spécifiques ("clinical benefit")**	☐	☐	
**Survie sans récidive**	☐	☐	
**Survie sans progression**	☐	☐	
**Taux de conservation sphinctérienne**	☐	☐	
**Survie sans symptôme**	☐	☐	
**Qualité de vie**	☐	☐	

Situation 22. Anus - métastatique

**Critère de substitution de la survie globale pour évaluer l'effet d'un traitement**	**Classement** Rang de pertinence (1, 2, 3 ...)	ou	*Ne pas étudier (cocher la case)*	**Précisions éventuelles**
**Survie sans progression**	☐		☐	
**Réponse**	☐		☐	
**Qualité de vie**	☐		☐	
**Contrôle de symptômes spécifiques ("clinical benefit")**	☐		☐	
**Survie sans progression + qualité de vie**	☐		☐	
**Survie sans symptôme**	☐		☐	
**Taux de conservation sphinctérienne**	☐		☐	
