# Supplementary material for: Surrogate endpoints for overall survival in digestive oncology trials: which candidates? A questionnaires survey among clinicians and methodologists
Source: BMC Cancer. 2010 Jun 10;10:277. doi: 10.1186/1471-2407-10-277 (PMC2904280; doi:10.1186/1471-2407-10-277)
Supplement: Additional file 3 — Propositions and rankings of potential surrogate endpoints for overall survival. In each situation, participants classified the surrogates from the most (#1) to the least relevant for surrogacy evaluation in their opinion, or checked "Do not study". Endpoints are classified according to the number of times they were chosen in first, second or third position. [file 1471-2407-10-277-S3.DOC]

| Situation | | Potential surrogate endpoint  (proposed by participants in the first questionnaire) | Number of participants  *Most Participant ranking Less*  *relevant (second questionnaire) relevant* | | | | | | | | | |  |  |
| --- | --- | --- | --- | --- | --- | --- | --- | --- | --- | --- | --- | --- | --- | --- |
|  |  |  | ***#1*** | ***#2*** | ***#3*** | ***#4*** | ***#5*** | ***#6*** | ***#7*** | ***#8*** | ***#9*** | ***"Do not study"*** | ***Missing*** | ***#1, #2,***  ***or #3***  ***n (%)*** |
| Oesophagus | neo-adjuvant treatment | Disease-free survival | 10 | 2 | 1 | 0 | 0 | 0 |  |  |  | 1 | 2 | 13 (81) |
|  |  | R0 resection | 1 | 3 | 7 | 0 | 1 | 0 |  |  |  | 1 | 3 | 11 (69) |
|  |  | Response | 3 | 2 | 2 | 3 | 1 | 0 |  |  |  | 2 | 3 | 7 (44) |
|  |  | Progression-free survival | 1 | 4 | 0 | 1 | 0 | 0 |  |  |  | 6 | 4 | 5 (31) |
|  |  | Early metabolic response | 1 | 2 | 2 | 4 | 3 | 0 |  |  |  | 1 | 3 | 5 (31) |
|  |  | Ratio involved nodes/ examined nodes | 0 | 0 | 1 | 2 | 1 | 3 |  |  |  | 6 | 3 | 1 (6) |
|  | non operable  non metastatic | Progression-free survival | 8 | 2 | 1 | 0 | 0 | 0 |  |  |  | 1 | 4 | 11 (69) |
|  |  | Response | 5 | 2 | 3 | 2 | 1 | 1 |  |  |  | 0 | 2 | 10 (63) |
|  |  | Disease-free survival | 2 | 3 | 3 | 0 | 1 | 1 |  |  |  | 2 | 4 | 8 (50) |
|  |  | Metastatic progression-free survival | 0 | 4 | 1 | 3 | 0 | 0 |  |  |  | 3 | 5 | 5 (31) |
|  |  | Metabolic response | 1 | 2 | 1 | 1 | 3 | 1 |  |  |  | 3 | 4 | 4 (25) |
|  |  | Dysphagia-free survival | 0 | 0 | 1 | 2 | 2 | 1 |  |  |  | 4 | 6 | 1 (6) |
|  | metastatic | Progression-free survival | 3 | 7 | 1 | 0 | 1 | 0 |  |  |  | 0 | 4 | 11 (69) |
|  |  | Quality of life + Progression-free survival | 8 | 2 | 0 | 0 | 1 | 0 |  |  |  | 0 | 5 | 10 (63) |
|  |  | Quality of life | 1 | 0 | 6 | 3 | 0 | 1 |  |  |  | 1 | 4 | 7 (44) |
|  |  | Response | 2 | 1 | 2 | 2 | 2 | 2 |  |  |  | 3 | 2 | 5 (31) |
|  |  | Clinical benefit | 0 | 2 | 3 | 3 | 2 | 0 |  |  |  | 0 | 6 | 5 (31) |
|  |  | Metabolic response | 0 | 1 | 1 | 1 | 2 | 1 |  |  |  | 5 | 5 | 2 (13) |
| Stomach | neo-adjuvant treatment | Disease-free survival | 13 | 1 | 1 | 0 |  |  |  |  |  | 0 | 1 | 15 (93) |
|  |  | R0 resection | 1 | 6 | 2 | 3 |  |  |  |  |  | 1 | 3 | 9 (56) |
|  |  | Response | 0 | 3 | 4 | 3 |  |  |  |  |  | 2 | 4 | 7 (44) |
|  |  | Progression-free survival | 1 | 3 | 2 | 0 |  |  |  |  |  | 5 | 5 | 6 (38) |
|  | metastatic | Quality of life + Progression-free survival | 7 | 5 | 1 | 0 | 0 |  |  |  |  | 0 | 3 | 13 (81) |
|  |  | Progression-free survival | 6 | 6 | 0 | 1 | 0 |  |  |  |  | 0 | 3 | 12 (75) |
|  |  | Response | 2 | 0 | 5 | 2 | 3 |  |  |  |  | 0 | 4 | 7 (44) |
|  |  | Quality of life | 0 | 1 | 6 | 4 | 1 |  |  |  |  | 2 | 2 | 7 (44) |
|  |  | Clinical benefit | 0 | 2 | 0 | 3 | 3 |  |  |  |  | 3 | 5 | 2 (13) |
| Liver | small CHC (Milan criteria) | Disease-free survival | 9 | 0 | 2 | 0 | 0 |  |  |  |  | 1 | 4 | 11 (69) |
|  |  | Progression-free survival | 3 | 3 | 1 | 0 | 0 |  |  |  |  | 5 | 4 | 7 (44) |
|  |  | Local control | 2 | 2 | 1 | 2 | 0 |  |  |  |  | 4 | 5 | 5 (31) |
|  |  | Response | 0 | 3 | 2 | 2 | 0 |  |  |  |  | 4 | 5 | 5 (31) |
|  |  | Hospitalisation-free survival | 0 | 1 | 1 | 1 | 2 |  |  |  |  | 6 | 5 | 2 (13) |
|  | big CHC (Milan criteria) | Progression-free survival | 13 | 0 | 1 | 0 | 0 | 0 |  |  |  | 0 | 2 | 14 (88) |
|  |  | Response | 0 | 6 | 2 | 1 | 0 | 0 |  |  |  | 2 | 5 | 8 (50) |
|  |  | Quality of life | 1 | 2 | 4 | 2 | 3 | 0 |  |  |  | 1 | 3 | 7 (44) |
|  |  | Hepato-cellular function | 0 | 2 | 1 | 2 | 2 | 3 |  |  |  | 1 | 5 | 3 (19) |
|  |  | Clinical benefit | 0 | 1 | 2 | 3 | 2 | 0 |  |  |  | 3 | 5 | 3 (19) |
|  |  | Hospitalisation-free survival | 0 | 2 | 1 | 2 | 1 | 1 |  |  |  | 5 | 4 | 3 (19) |
|  | metastatic | Quality of life + Progression-free survival | 9 | 3 | 1 | 0 | 0 | 0 | 0 |  |  | 0 | 3 | 13 (81) |
|  |  | Progression-free survival | 4 | 3 | 2 | 1 | 0 | 0 | 0 |  |  | 2 | 4 | 9 (56) |
|  |  | Quality of life | 0 | 3 | 2 | 2 | 3 | 0 | 0 |  |  | 1 | 5 | 5 (31) |
|  |  | Hospitalisation-free survival | 1 | 0 | 1 | 2 | 0 | 1 | 1 |  |  | 5 | 5 | 2 (13) |
|  |  | Response | 0 | 1 | 1 | 3 | 1 | 2 | 0 |  |  | 3 | 5 | 2 (13) |
|  |  | Clinical benefit | 0 | 0 | 2 | 1 | 5 | 1 | 0 |  |  | 2 | 5 | 2 (13) |
|  |  | Alpha FP Kinetic | 0 | 1 | 1 | 1 | 0 | 4 | 3 |  |  | 2 | 4 | 2 (13) |

| Situation | | Potential surrogate endpoint  (proposed by participants in the first questionnaire) | Number of participants  *Most Participant ranking Less*  *relevant (second questionnaire) relevant* | | | | | | | | | |  | |  |
| --- | --- | --- | --- | --- | --- | --- | --- | --- | --- | --- | --- | --- | --- | --- | --- |
|  |  |  | ***#1*** | ***#2*** | ***#3*** | ***#4*** | ***#5*** | ***#6*** | ***#7*** | ***#8*** | ***#9*** | ***"Do not study"*** | ***Missing*** | | ***#1, #2,***  ***or #3***  ***n (%)*** |
| Pancreas | neo-adjuvant treatment | Disease-free survival | 11 | 2 | 1 | 0 | 0 |  |  |  |  | 0 | | 2 | 14 (88) |
|  |  | R0-1 resection | 2 | 9 | 1 | 1 | 0 |  |  |  |  | 1 | | 2 | 12 (75) |
|  |  | Progression-free survival | 2 | 1 | 2 | 0 | 0 |  |  |  |  | 6 | | 5 | 5 (31) |
|  |  | Response | 0 | 0 | 5 | 1 | 0 |  |  |  |  | 5 | | 5 | 5 (31) |
|  |  | Hospitalisation-free survival | 0 | 1 | 1 | 1 | 1 |  |  |  |  | 7 | | 5 | 2 (13) |
|  | non operable | Progression-free survival | 14 | 0 | 0 | 0 | 0 | 0 | 0 |  |  | 0 | | 2 | 14 (88) |
|  |  | Quality of life | 0 | 6 | 2 | 2 | 3 | 1 | 0 |  |  | 0 | | 2 | 8 (50) |
|  |  | Metastatic progression-free survival | 0 | 5 | 2 | 0 | 3 | 0 | 0 |  |  | 1 | | 5 | 7 (44) |
|  |  | Response | 1 | 3 | 2 | 2 | 0 | 2 | 0 |  |  | 2 | | 4 | 6 (38) |
|  |  | Disease-free survival | 0 | 0 | 2 | 0 | 0 | 1 | 0 |  |  | 9 | | 4 | 2 (13) |
|  |  | Clinical benefit | 0 | 0 | 2 | 6 | 2 | 0 | 0 |  |  | 1 | | 5 | 2 (13) |
|  |  | Hospitalisation-free survival | 0 | 0 | 2 | 0 | 0 | 1 | 1 |  |  | 7 | | 5 | 2 (13) |
|  | metastatic | Progression-free survival | 10 | 3 | 0 | 0 | 0 | 1 | 0 | 0 |  | 0 | | 2 | 13 (81) |
|  |  | Symptom-free survival | 2 | 4 | 2 | 1 | 0 | 0 | 0 | 0 |  | 3 | | 4 | 8 (50) |
|  |  | Response | 0 | 4 | 3 | 1 | 3 | 0 | 0 | 0 |  | 3 | | 2 | 7 (44) |
|  |  | Clinical benefit | 1 | 1 | 4 | 3 | 0 | 1 | 0 | 0 |  | 1 | | 5 | 6 (38) |
|  |  | Quality of life | 1 | 1 | 3 | 5 | 3 | 0 | 0 | 0 |  | 1 | | 2 | 5 (31) |
|  |  | CA19-9 decline >50% | 0 | 0 | 1 | 0 | 0 | 0 | 3 | 1 |  | 6 | | 5 | 1 (6) |
|  |  | Hospitalisation-free survival | 0 | 1 | 0 | 0 | 0 | 3 | 0 | 0 |  | 7 | | 5 | 1 (6) |
|  |  | Pain | 0 | 0 | 1 | 0 | 2 | 1 | 2 | 1 |  | 4 | | 5 | 1 (6) |
| Biliary tract | neo-adjuvant treatment | Disease-free survival | 11 | 2 | 0 | 0 | 0 |  |  |  |  | 0 | | 3 | 13 (81) |
|  |  | R0 resection | 0 | 8 | 2 | 1 | 0 |  |  |  |  | 1 | | 4 | 10 (63) |
|  |  | Progression-free survival | 1 | 2 | 1 | 0 | 0 |  |  |  |  | 6 | | 6 | 4 (25) |
|  |  | Hospitalisation-free survival | 0 | 1 | 1 | 1 | 0 |  |  |  |  | 7 | | 6 | 2 (13) |
|  |  | Icterus-free survival | 0 | 0 | 1 | 2 | 2 |  |  |  |  | 5 | | 6 | 1 (6) |
|  | non operable  non metastatic | Progression-free survival | 12 | 1 | 0 | 0 | 0 |  |  |  |  | 0 | | 3 | 13 (81) |
|  |  | Quality of life | 1 | 2 | 7 | 1 | 0 |  |  |  |  | 1 | | 4 | 10 (63) |
|  |  | Response | 0 | 7 | 2 | 0 | 1 |  |  |  |  | 3 | | 3 | 9 (56) |
|  |  | Icterus-free survival | 0 | 1 | 1 | 0 | 2 |  |  |  |  | 6 | | 6 | 2 (13) |
|  |  | Hospitalisation-free survival | 0 | 1 | 0 | 3 | 0 |  |  |  |  | 6 | | 6 | 1 (6) |
|  | metastatic | Progression-free survival | 11 | 0 | 1 | 1 | 0 |  |  |  |  | 0 | | 3 | 12 (75) |
|  |  | Response | 1 | 8 | 0 | 1 | 1 |  |  |  |  | 2 | | 3 | 9 (56) |
|  |  | Quality of life | 1 | 1 | 6 | 4 | 0 |  |  |  |  | 0 | | 4 | 8 (50) |
|  |  | Clinical benefit | 0 | 3 | 4 | 2 | 0 |  |  |  |  | 1 | | 6 | 7 (44) |
|  |  | Hospitalisation-free survival | 0 | 1 | 0 | 0 | 3 |  |  |  |  | 6 | | 6 | 1 (6) |
| Digestive lymphoma | Localized | Disease-free survival | 9 | 0 | 2 | 0 | 0 | 0 |  |  |  | 0 | | 5 | 11 (69) |
|  |  | Progression-free survival | 2 | 3 | 0 | 0 | 0 | 0 |  |  |  | 4 | | 7 | 5 (31) |
|  |  | Response | 1 | 4 | 0 | 1 | 2 | 0 |  |  |  | 1 | | 7 | 5 (31) |
|  |  | Percentage of high-grade transformation per year | 0 | 2 | 2 | 1 | 1 | 0 |  |  |  | 1 | | 9 | 4 (25) |
|  |  | Gastrectomy avoidance | 0 | 0 | 2 | 2 | 0 | 2 |  |  |  | 3 | | 7 | 2 (13) |
|  |  | Quality of life | 0 | 0 | 1 | 3 | 2 | 1 |  |  |  | 2 | | 7 | 1 (6) |
|  | metastatic | Disease-free survival | 5 | 2 | 3 | 1 |  |  |  |  |  | 1 | | 4 | 10 (63) |
|  |  | Progression-free survival | 5 | 4 | 0 | 1 |  |  |  |  |  | 0 | | 6 | 9 (56) |
|  |  | Response | 1 | 2 | 3 | 3 |  |  |  |  |  | 0 | | 7 | 6 (38) |
|  |  | Time-to-remission | 1 | 2 | 3 | 2 |  |  |  |  |  | 1 | | 7 | 6 (38) |

| Situation | | Potential surrogate endpoint  (proposed by participants in the first questionnaire) | Number of participants  *Most Participant ranking Less*  *relevant (second questionnaire) relevant* | | | | | | | | | |  | |  |
| --- | --- | --- | --- | --- | --- | --- | --- | --- | --- | --- | --- | --- | --- | --- | --- |
|  |  |  | ***#1*** | ***#2*** | ***#3*** | ***#4*** | ***#5*** | ***#6*** | ***#7*** | ***#8*** | ***#9*** | ***"Do not study"*** | ***Missing*** | | ***#1, #2,***  ***or #3***  ***n (%)*** |
| Colon | adjuvant treatment | Disease-free survival | 13 | 0 | 2 | 0 |  |  |  |  |  | 1 | | 0 | 15 (93) |
|  |  | Specific survival | 1 | 6 | 2 | 0 |  |  |  |  |  | 4 | | 3 | 9 (56) |
|  |  | Quality of life | 0 | 1 | 4 | 2 |  |  |  |  |  | 5 | | 4 | 5 (31) |
|  |  | Progression-free survival | 1 | 3 | 0 | 1 |  |  |  |  |  | 8 | | 3 | 4 (25) |
| Rectum | neo-adjuvant treatment | Disease-free survival | 13 | 1 | 2 | 0 | 0 |  |  |  |  | 0 | | 0 | 16 (100) |
|  |  | Response | 2 | 6 | 3 | 1 | 1 |  |  |  |  | 1 | | 2 | 11 (69) |
|  |  | Complete resection | 1 | 5 | 3 | 2 | 0 |  |  |  |  | 2 | | 3 | 9 (56) |
|  |  | Sphincter preservation | 0 | 0 | 3 | 4 | 3 |  |  |  |  | 3 | | 3 | 3 (19) |
|  |  | Quality of life | 0 | 0 | 1 | 3 | 6 |  |  |  |  | 3 | | 3 | 1 (6) |
| Colon-rectum | metastatic | Progression-free survival | 7 | 4 | 1 | 1 | 0 | 0 | 0 | 0 | 0 | 0 | | 3 | 12 (75) |
|  |  | Quality of life + Progression-free survival | 6 | 1 | 2 | 0 | 1 | 1 | 0 | 0 | 0 | 0 | | 5 | 9 (56) |
|  |  | R0 metastatic resection rate | 2 | 3 | 4 | 2 | 1 | 0 | 1 | 0 | 0 | 1 | | 2 | 9 (56) |
|  |  | Response | 1 | 1 | 2 | 3 | 0 | 2 | 1 | 0 | 0 | 1 | | 5 | 4 (25) |
|  |  | Quality of life | 0 | 2 | 1 | 1 | 0 | 4 | 0 | 1 | 0 | 1 | | 6 | 3 (19) |
|  |  | Ratio overall survival / chemotherapy duration | 0 | 2 | 1 | 2 | 2 | 0 | 0 | 1 | 0 | 3 | | 5 | 3 (19) |
|  |  | Cumulative time without cytotoxic treatment | 0 | 2 | 0 | 0 | 2 | 0 | 1 | 1 | 1 | 4 | | 5 | 2 (13) |
|  |  | Maintenance regimen-free survival | 0 | 0 | 1 | 1 | 2 | 0 | 2 | 1 | 0 | 4 | | 5 | 1 (6) |
|  |  | Early tumour markers measurement at 1 month | 0 | 0 | 0 | 0 | 0 | 1 | 1 | 0 | 1 | 8 | | 5 | 0 |
| Anus | localized | Disease-free survival | 11 | 0 | 0 | 1 | 0 |  |  |  |  | 0 | | 4 | 11 (69) |
|  |  | Response | 1 | 5 | 2 | 2 | 1 |  |  |  |  | 1 | | 4 | 8 (50) |
|  |  | Sphincter preservation rate | 0 | 4 | 4 | 1 | 0 |  |  |  |  | 1 | | 6 | 8 (50) |
|  |  | Abdominoperitoneal amputation-free survival | 0 | 2 | 3 | 2 | 1 |  |  |  |  | 2 | | 6 | 5 (31) |
|  |  | Progression-free survival | 2 | 1 | 0 | 0 | 1 |  |  |  |  | 6 | | 6 | 3 (19) |
|  | locally advanced | Disease-free survival | 4 | 3 | 1 | 1 | 0 | 1 | 1 | 0 |  | 1 | | 4 | 8 (50) |
|  |  | Progression-free survival | 5 | 2 | 0 | 1 | 0 | 0 | 0 | 0 |  | 2 | | 6 | 7 (44) |
|  |  | Response | 2 | 2 | 1 | 0 | 3 | 0 | 0 | 1 |  | 0 | | 7 | 5 (31) |
|  |  | Abdominoperitoneal amputation-free survival | 1 | 1 | 2 | 2 | 1 | 0 | 0 | 1 |  | 1 | | 7 | 4 (25) |
|  |  | Sphincter preservation rate | 0 | 1 | 3 | 1 | 0 | 0 | 1 | 1 |  | 2 | | 7 | 4 (25) |
|  |  | Symptom-free survival | 0 | 1 | 1 | 0 | 0 | 4 | 0 | 0 |  | 3 | | 7 | 2 (13) |
|  |  | Clinical benefit | 0 | 0 | 1 | 2 | 2 | 0 | 1 | 1 |  | 1 | | 8 | 1 (6) |
|  |  | Quality of life | 0 | 0 | 0 | 1 | 1 | 1 | 3 | 1 |  | 2 | | 7 | 0 |
|  | metastatic | Progression-free survival | 6 | 4 | 1 | 1 | 0 | 0 | 0 |  |  | 0 | | 4 | 11 (69) |
|  |  | Quality of life + Progression-free survival | 7 | 1 | 1 | 0 | 0 | 0 | 0 |  |  | 0 | | 7 | 9 (56) |
|  |  | Symptom-free survival | 0 | 3 | 2 | 0 | 2 | 0 | 0 |  |  | 2 | | 7 | 5 (31) |
|  |  | Response | 0 | 0 | 4 | 2 | 1 | 1 | 1 |  |  | 1 | | 6 | 4 (25) |
|  |  | Quality of life | 0 | 3 | 1 | 1 | 1 | 3 | 1 |  |  | 1 | | 5 | 4 (25) |
|  |  | Clinical benefit | 0 | 0 | 0 | 2 | 2 | 1 | 2 |  |  | 2 | | 7 | 0 |
|  |  | Sphincter preservation rate | 0 | 0 | 0 | 2 | 1 | 2 | 2 |  |  | 2 | | 7 | 0 |
